# Development and Validation of Internet of Things-Enabled Weighing System for Cage-Free Poultry Houses

**DOI:** 10.3390/s26041279

**Published:** 2026-02-16

**Authors:** Anjan Dhungana, Bidur Paneru, Samin Dahal, Zhihang Song, Lilong Chai

**Affiliations:** 1 Department of Poultry Science, University of Georgia, Athens, GA 30602, USA; 2 Department of Horticulture, University of Georgia, Athens, GA 30602, USA

**Keywords:** automated assessment, body weight, poultry farming, poultry welfare monitoring, wireless data acquisition

## Abstract

Accurate body-weight monitoring is essential for assessing welfare in cage-free poultry. However, commercial farms continue to rely on manual weighing because of concerns regarding the accuracy and reliability of automated methods. This study developed and evaluated an Internet of things (IoT)-enabled weighing platform integrating load cells, an microcontroller, a Raspberry Pi 5, and Node-RED for data acquisition, processing, and visualization. The system recorded weight measurements at 1 Hz, detected individual weighing sessions, and applied a rolling-median filter to produce stable weight estimates. Validation was performed against a reference scale during two weighing sessions one week apart using 75 cage-free hens randomly selected from a flock of 750 Hy-Line W80 birds. Bland–Altman analysis and a linear mixed-effects model indicated a small overestimation of approximately 6–9 g, with most measurements falling within the 95% limits of agreement, while overall mean absolute percentage error remained below 3%. Improved accuracy during the second session suggests that platform stability influenced performance. Overall, the system demonstrates strong potential for continuous low-stress weight monitoring in poultry farms. Future improvements should focus on refining calibration methods, enhancing mechanical stability, and integrating bird identification and presence-detection mechanisms to further support flock management and welfare monitoring.

## 1. Introduction

Poultry welfare monitoring is an important aspect of modern animal husbandry, particularly in cage-free layer, broiler, and breeder production systems, where birds have greater freedom of movement but are also exposed to a broader range of health risks. Body weight is one of the most important welfare indicators for cage-free layers or pullets (young hens < 17 weeks), which reflects growth rates, overall health and may point out potential welfare issues in a poultry house. In layer hens/pullets, body weight is associated with the timely onset of sexual maturity and egg production [1,2]. Egg quality attributes, including the egg size, egg weight, and shell strength, are closely linked to the body weight of laying hens. Studies have shown that hens with lower body weights tend to produce smaller and lighter eggs compared to the heavier birds [3]. Conversely, excessive body weight has been associated with reductions in shell strength and egg shape index, which can lead to a higher incidence of cracked eggs and reduced market value [4]. Maintaining an appropriate body weight and flock weight uniformity is therefore important for sustaining high and consistent egg production throughout the laying cycle [2].

In cage-free systems, hens typically exhibit a lower body weight than in the caged systems due to higher physical activity [5]. As a result, the frequent monitoring of flock weight becomes an essential management practice to ensure proper growth, optimize performance, and support welfare. The most common technique for checking the flock uniformity in cage-free flocks is to randomly sample and weigh around 2% of the birds from the flock and place the chickens over a weighing machine until a stable weight is reached [6].

The traditional method of weight measurement systems, however, presents several limitations. In commercial trials, manual sampling is carried out weekly for performance monitoring and sometimes more frequently for closely tracking growth [7]. This process is labor-intensive, time-consuming and requires handling of birds, which can induce stress and disrupt natural behavior, with potential negative effects on welfare and productivity [6,8]. Previous studies have shown that repeated exposure to handling-related stressors may impair the long-term stress-coping ability of laying hens [6,9]. Such stress exposure has also been associated with adverse welfare outcomes later in life, including increased comb and wattle injuries as well as more feather damage [8,10]. Additionally, sampling bias is a major concern in manual weighing in cage-free systems, as the handlers tend to catch the heavier and docile birds more than the agile birds, leading to the overestimation of the body weights [7].

These challenges make manual weighing less practical for high-frequency monitoring, especially in large cage-free barns where bird movement is dynamic and difficult to control. Recent advancements have explored the automation of this process using physical sensor-based scales and computer vision-based solutions utilizing 2D or 3D cameras with deep learning models [6,9,10,11]. However, most of the explored solutions either require complex setups or have limited compatibility with commercial farm conditions. For example, computer vision-based systems such as the ones used by Mortensen et al. [12] uses 3D cameras connected to computing system paired with artificial neural network, which may require farm-specific calibration and retraining due to variations in barn layout, lighting, and bird density, limiting their scalability and ease of deployment in commercial settings.

Although the importance of continuous and accurate body weight monitoring in cage-free production is well established, there remains a need for a low-cost, reliable and practical solution requiring minimal human intervention. Accordingly, there is a need to develop a sensor-based automated weighing system that operates reliably under cage-free conditions, minimizes human handling and resulting stress, ensures accuracy comparable to manual weighing, and continuously logs weight information for remote access.

This study addresses this gap by designing, building, and evaluating a weighing system integrated with ESP32-based load-cell platform, data filtering algorithm, and data transmission protocol, with the goal of providing real-time flock-level weight monitoring in cage-free poultry production systems.

## 2. Materials and Methods

The study was conducted in an experimental cage-free layer facility at the University of Georgia, Athens, housing six weeks old 750 Hy-Line W80 laying hens. The facility consisted of three cage-free rooms, each measuring 7.3 m × 6.1 m × 3.0 m (L × W × H). The animal use and management procedures were approved by the Institutional Animal Care and Use Committee (IACUC) of the University of Georgia (AUP# A2023 02-024-Y3-A1).

The methodology for developing and evaluating the automated weighing system consisted of (1) system design and hardware assembly, (2) data acquisition and transmission, (3) data filtering, logging and communication, and (4) validation experiments to compare with the standard weighing system.

### 2.1. System Design and Hardware Assembly

**Figure 1:**
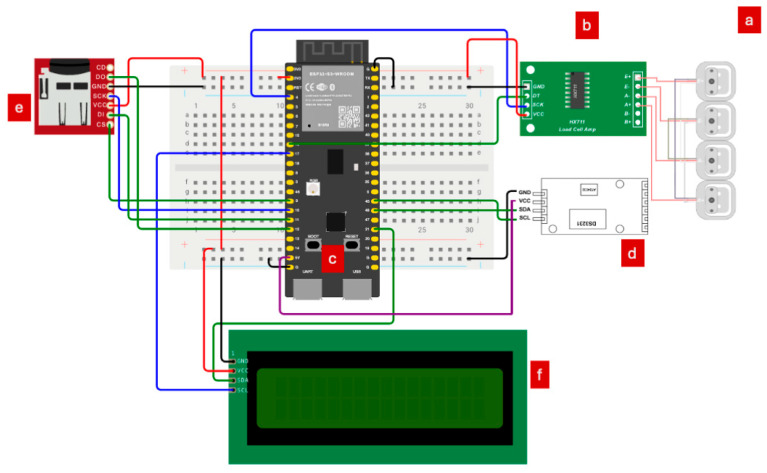
Wiring diagram for the weight detection system. (**a**) Four load cells, (**b**) HX711 amplifier, (**c**) a ESP32-S3 microcontroller unit, (**d**) Real-time clock module, (**e**) SD card module, and (**f**) I^2^C LCD screen.

As shown in the wiring diagram in Figure 1, the weighing system was built using (a) four load cells, (b) an HX711 amplifier (Avia Semiconductor Ltd., Xiamen, China), an analog-to-digital converter (ADC) that converts analog load sensors readings to digital signals and sends it to the microcontroller unit (henceforth MCU), and (c) an ESP32-S3 MCU (Espressif systems, Sanghai, China). This MCU was chosen due to its integrated Wi-Fi and Bluetooth capabilities without the need for external communication modules. The additional components included a Real-time clock (RTC) module (Analog Devices, Inc., Wilmington, MA, USA) (d) for accurate timestamping, an SD card module (e) for local data storage, an I^2^C LCD screen (f) for on-device monitoring, and an external battery pack for power supply to the device. Additional to the MCU, a single board computer—Raspberry Pi 5 (Raspberry Pi Ltd., Cambridge, UK)—was added to the system for wireless data transmission, data processing, storage and visualization using user interface.

The MCU performed four major functions:

1. Measurement: Read calibrated weight values (grams) from the load-cell system at a sampling rate of 1 Hz.
2. Session detection: Identify individual bird visits by applying a predefined weight threshold to distinguish when a chicken stepped onto or off the platform.
3. Data logging: Save all raw weight measurements, along with their timestamps and session identifiers, to an onboard SD card in comma-separated values (CSV) format.
4. Wireless transmission: Publish each measurement as a CSV-formatted MQTT message to a Raspberry Pi 5 for real-time processing, storage, and visualization.

The components used in this weighing system were low-cost and were acquired for around 118 USD. The cost breakdown for the components required to build a similar system are provided below in Table 1.

### 2.2. Data Acquisition and Transmission

#### 2.2.1. Calibration and Bench Testing

The load sensors were placed underneath a PVC board measuring 28 × 35 cm such that it transmits all the loads to the sensors equally. Before deployment, the weighing platform was calibrated using a standard reference weight measuring 500 g. The calibration factor was computed as the ratio between the raw HX711 reading and the known reference weight (Equation (1)), and this factor was subsequently used to convert the raw sensor readings to values in grams during data collection (Equation (2)). Since the raw output differed slightly each time when known weights were placed over the platform, 5 readings were taken and averaged to input as ‘reading’ in Equation (1).

(1) $$Calibration\;factor=\frac{HX711\;reading}{known\;weight}$$

(2) $$Weight\;\left(grams\right)=\frac{HX711\;reading}{calibration\;factor}$$

This calibration approach was selected to maintain simplicity and reproducibility for a low-cost system. However, this method assumes a linear sensor response and does not account for potential non-linear behavior or uneven load distribution caused by bird posture and movement.

#### 2.2.2. Data Acquisition

Weights were sampled at 1 Hz and converted to grams using the calibrated load-cell equation. During calibration, small noise readings (<100 g) appeared even when the platform was empty; so, a threshold logic was applied: a session began only when the measured weight was ≥100 g. Once a bird stepped onto the platform, the system recorded up to 10 readings per session to avoid oversampling and to reduce the variability caused by movement. A session ended when the weight dropped below the threshold, and a new session began when the threshold was crossed again.

For each session, a unique measurement id was assigned, which incremented sequentially and was stored in an SD card to prevent ID duplication in case of power loss. The measurements were stored as CSV files with the following header in the SD card.

date, time, unix_time, weight, measurement_id

Under the headings mentioned above, date, time, and UNIX time were provided by the RTC module. The MCU used the current date and time provided by the RTC module and appended the data with weight and the measurement id. Accurate timestamping ensured time-based ordering of the sample weights and ensured time-based statistics can be extracted using the stored information in the future.

#### 2.2.3. Data Transmission

The raw data consisting of all the measurements were saved in the SD card and simultaneously transmitted to the Raspberry pi 5 (Rpi) over Wi-Fi using Message Queuing Telemetry Transport (MQTT) protocol (MQTT 3.1.1). The Rpi was configured as an MQTT broker, and the MCU was set up as a publisher. With the start of each session, each row consisting of headers date, time, UNIX time, weight, and measurement id were transmitted using topic ‘weightlogger/weights’, such that the MQTT payload exactly matched the SD card format.

After each session was over, a python script (Python 3.11) running in the Rpi performed data filtering to get a stable weight.

### 2.3. Data Filtering

Data filtering was performed on the Rpi to eliminate variations due to constant movement and electronic noises. After wireless data acquisition from MCU and the completion of a session, filtering was applied to each session by the Rpi using subscriber python script. A session was considered complete when either of the following conditions were satisfied:

i. The Rpi did not receive data for more than 3 s.
ii. The MCU ended a session because weight dropped below the threshold or had 10 data points for a single session.

The first step was to apply rolling median smoothing using a 3-point rolling median filter. A rolling median filter or moving median filter has been previously used in processing raw signals and provides an advantage over other methods such as mean filtering to smooth noisy time-series data [13].

This can be represented by Equation (3):

(3) $$w_{filtered}(i)=median(w_{i-1},w_{i},w_{i+1})$$

where $w_{i}$ is the raw weight reading at time I, and $w_{filtered}(i)$ is the smoothed weight.

This filter was applied, as it removes sudden spikes and is robust enough to remove outliers, due to dynamic objects like hens, which have constant movement.

After median smoothing, the median of the filtered values was used to estimate the final weight. This can be represented by Equation (4).

(4) $$w_{final}=median(w_{filtered}\left(1\right),w_{filtered}\left(2\right),\ldots ,w_{filtered}(n))$$

**Figure 2:**
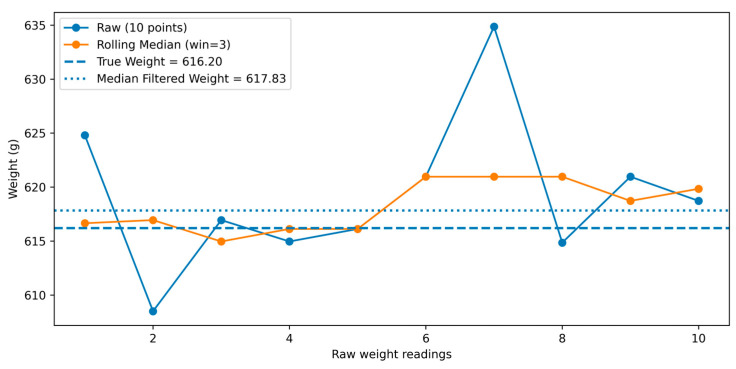
Data filtering technique using rolling median smoothing and median filtering. The rolling median smoothing removes sudden spikes from a series of data points.

After the filtering was complete and final weight was acquired, the Rpi saved the weights into new csv file under the headings: measurement_id, number of samples, start timestamp, end timestamp, median of filtered weights. An example of data filtering is shown in Figure 2.

**Figure 3:**
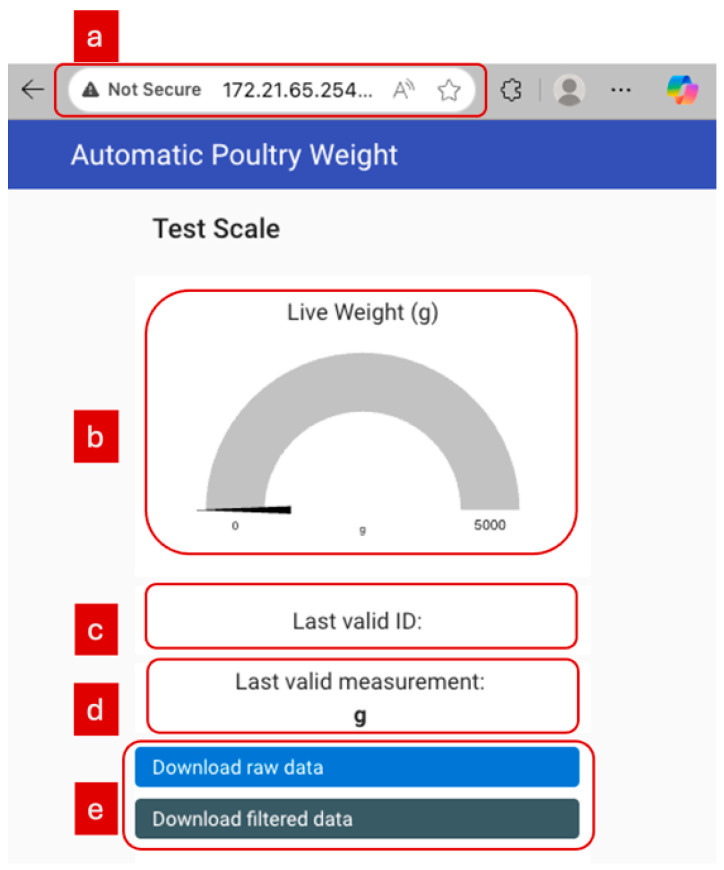
Node-RED dashboard for visualization of the bird weights. (**a**) Remote connection to the dashboard for wireless data visualization. (**b**) Live weight gauge. (**c**) Last valid id for the weight measurement. (**d**) Last valid weight for the corresponding valid id. (**e**) Download buttons for the raw and filtered data.

The session ID and the filtered weights were published over MQTT to the Node-RED dashboard under the topics ‘weightlogger/last_valid_id’ and ‘weightlogger/last_valid’ for display. In summary, the MCU acted to acquire calibrated weights, store data locally, and transmit the raw data streams to the Rpi, which acted as a processing unit to perform filtering and storage, and Node-RED performed real-time data visualization. The visualization dashboard for live weight monitoring is shown in Figure 3.

### 2.4. Validation Experiment

**Figure 4:**
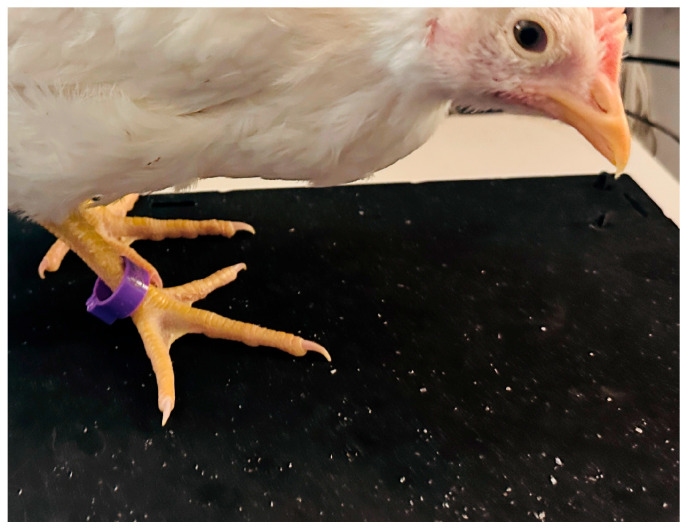
Leg band on a sample bird (purple plastic band).

For the validation experiment, 75 birds were randomly selected from a flock of 750 birds housed in 3 different cage-free research spaces (25 birds from one space). Each of the birds were tagged with uniquely numbered leg bands as shown in Figure 4. Weight data were taken twice, one week apart using a standard scale (Ohaus Ranger 3000 bench scale; Parsippany, NJ, USA) and the developed measurement system. Taking two weight data for a single bird allowed to analyze the impact of different weight levels and weighing methods on the measurement accuracy.

**Figure 5:**
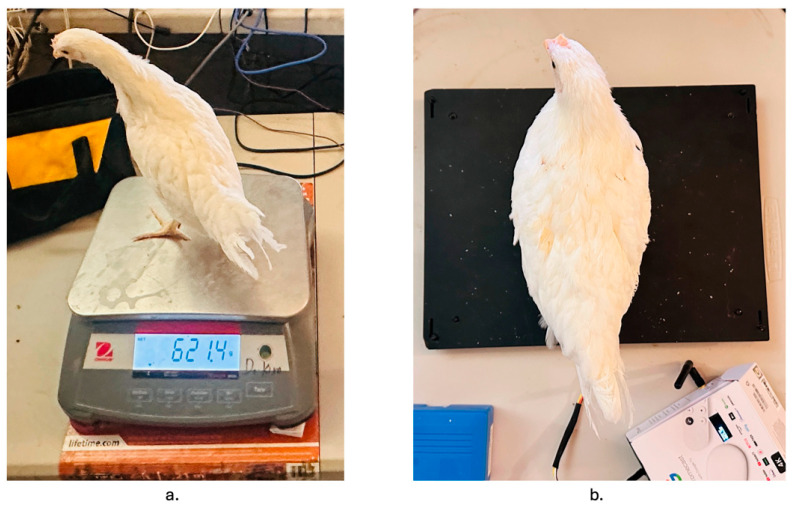
(**a**) Weight measurement using standard scale; (**b**) weight measurement using the developed scale. Both scales were placed side by side on a flat surface.

**Figure 6:**
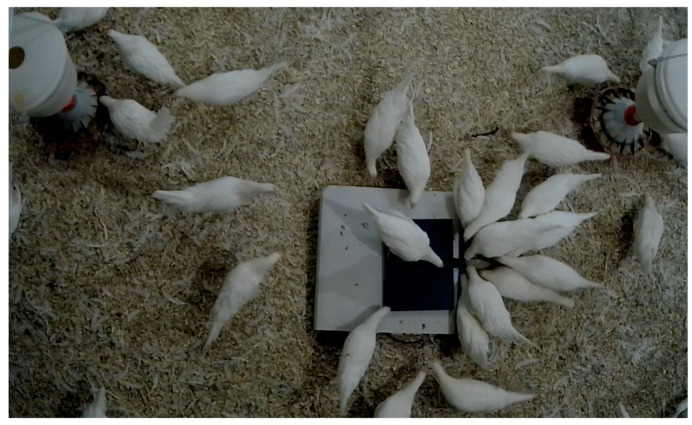
Placement of the weighing platform inside poultry house.

To know the exact differences between weight of each bird, they were manually placed on both standard (Figure 5a) and manual scale (Figure 5b). The readings from standard scale, leg band IDs, and the corresponding ‘Last valid ID’ shown by the dashboard were recorded manually for future analysis. After the validation experiment was complete, the platform was placed inside the cage-free research house as shown in Figure 6. The platform was placed on a flat surface, approximately 1.5 m from the feeder in an open area.

### 2.5. Data Analysis

The data obtained from the two weighing methods were statistically analyzed using Python. Data processing was carried out using the Pandas package (version 2.1), and statistical tests were performed using the SciPy Statistics module (version 1.1). Visualization of the results was created using Matplotlib (version 3.7) and Seaborn (version 0.12). To ensure error free analysis, a unique id was assigned to each bird by concatenating the cage-free space number and the bird id (formatted as ‘room_birdID’). Additionally, any rows and the corresponding measurement on another date with missing measurements were removed. Missing values resulted from the researchers’ inability to identify the same individual birds across the two measurement dates. After filtering, 138 measurements from 69 unique birds remained for analysis.

To evaluate the degree of agreement between the two measurement methods, Bland–Altman analysis was used [14]. This analysis calculates the difference between the two methods for each observation and plots these differences against the mean of two measurements. The mean difference, also known as bias, indicates if there is an overestimation or underestimation of the weight by one system compared to the other. The positive bias indicates that the system overestimates the weight on average, while negative bias indicates the underestimation. Additionally, for the same analysis, limits of agreement (LOA) were calculated using Equation (5).

(5) LOA=bias±1.96×SD

This equation is used to calculate the range in which 95% of the measurements are statistically expected to fall. A narrow LOA indicates strong agreement between two different methods, while wider limits suggest variability. The factor 1.96 in Equation (5) is derived from the normal distribution and represents the interval that captures approximately 95% of observations when measurement differences are assumed to be normally distributed [14].

Since each the measurements were taken on two different dates for same birds, the analysis required a repeated-measures Bland–Altman approach, which accounts for multiple measurements collected from the same individual. This was implemented using a mixed-effects model, where bird identity was treated as a random effect. The model can be represented by Equation (6).

(6) $$difference_{ij}=\beta_{0}+u_{i}+\epsilon_{ij}$$

where $\beta_{0}$ is overall mean bias;

$u_{i}$ represents random effect for bird ‘i’;

$\epsilon_{ij}$ represents residual error.

For the sensor-based system, the output used in the analysis was the median filtered weight, derived using a 3-point rolling median filter. This filtering method removes momentary fluctuations caused by bird movement and provides a more stable estimate of the bird’s true body weight.

In addition to the Bland–Altman analysis, the mean absolute percentage error (MAPE) was calculated to quantify the accuracy of the developed system. The MAPE was calculated as shown in Equation (7).

(7) $$MAPE=\frac{1}{n}\sum_{i=1}^{n}\left|\frac{W_{sensor,i}-W_{reference,i}}{W_{reference,i}}\right|\times 100$$

Additional analyses were done to complement the Bland–Altman analysis. This included the correlation coefficient to quantify the linear association between the two methods, mean absolute differences and summary statistics to compare the distribution of weights across two measurement methods.

## 3. Results and Discussion

**Figure 7:**
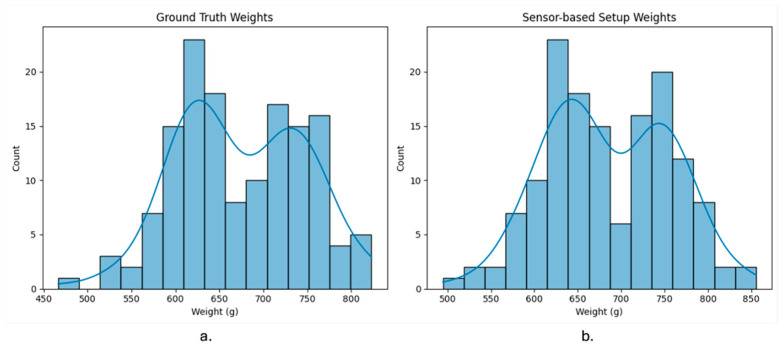
Distribution of bird weights measured using standard scale (**a**) and the sensor-based system (**b**). The sensor-based system captures the overall shape of the distribution but shows a rightward shift, indicating overestimation compared to the standard scale.

**Figure 8:**
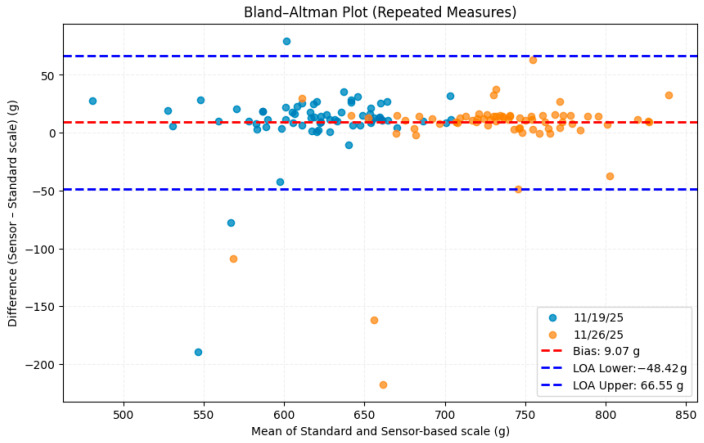
Bland–Altman analysis showing the level of agreement between sensor-based and standard measurement system on two different dates. The highest and lowest dotted lines represent the upper and lower limits of agreement, respectively, and the dotted line in the middle shows the mean bias between two measurement methods.

Descriptive statistics of the ground-truth measurements indicated that birds weighed an average of 615.78 g during the first weighing and 733.18 g during the second weighing, with corresponding standard deviations of 41.07 g and 46.43 g. Visualization of the weight distributions from both the standard scale and the sensor-based system (Figure 7) shows that the automated platform closely reproduces the overall shape and variability of the flock’s weight distribution. However, the sensor-based histogram is slightly shifted to the right, suggesting a tendency for the system to overestimate weights relative to the reference scale. This trend is further supported by the Bland–Altman analysis (Figure 8) and the linear mixed-effects model, both of which identified a small positive bias in the sensor-based measurements.

The MAPE was calculated separately for each weighing date to quantify the average percentage deviation of the sensor-based system from the standard scale. The system achieved a MAPE of 3.16% (accuracy = 96.84%) on the first weighing date, which improved to 2.74% (accuracy = 97.05%) on the second date, demonstrating increased measurement consistency. This improvement is likely due to relocating the weighing platform onto a more stable surface, which reduced movement-related noise and provided more uniform load distribution across the sensors. Further analysis of agreement between the two weighing methods supports this trend and is presented in later sections.

The Bland–Altman analysis (Figure 8) yielded the mean bias (sensor based − standard) of 9.07 g, indicating a small overestimation by the automated system relative to the standard scale. Plotting the mean of two measurements against their difference showed that majority of the measurements clustered around zero, demonstrating a good agreement across most of the birds. However, some outliers were observed, mostly due to the birds only staying on the platform momentarily resulting in unstable weights.

Since Bland–Altman analysis only provides an arithmetic measure, a mixed-effects model was used to calculate the adjusted bias. The adjusted mean bias was 6.63 g (SE = 3.20, *p* = 0.038), confirming the earlier analyses showing that the sensor-based system overestimated in comparison to the standard scale. The random-effect variance (181.80 g^2^; SD ≈ 13.48 g) indicated that the birds differed in their average measurement bias. This between-subject variability reflects individual differences on the weighing platform. This variability may have resulted from different bird movements, leg positions and their posture.

Compared with the commercial automated weighing system reported by Zhou et al. [6], which showed an average error of 10.3 g, the system developed in this study demonstrated a lower bias (6.63 g). However, their system achieved a lower MAPE (0.5%) and a smaller standard deviation of percentage error (2.3%) than those observed in our study (3.16% and 5.12%, respectively). This indicates that despite the developed system producing a lower mean error than the commercial system, its higher MAPE and larger variability means that the commercial system delivers more consistent and stable weight estimates across birds.

In our study, we did not analyze the bird–platform interaction as it was beyond the scope the current work. However, several behavioral factors may significantly influence weight estimations. For example, Chedad et al. [15] found that lighter broilers tend to use the automatic weighing platforms more frequently than the heavier birds. Although direct evidence for similar behavior in laying hens is limited, it can be assumed that heavier birds, due to their lower locomotive activities [16], may access the weighing platform less frequently than the lighter birds. This could lead to underestimations of the true weights.

The results of the present study showed a consistent overestimation of body weight by the developed system. Although the exact source of this bias could not be conclusively identified, several factors may have contributed. One likely explanation is the simple linear calibration procedure that was used to convert the raw sensor readings into weight values, as this may not fully account for a potential non-linear response across measurements, uneven load distribution on the platform, or mechanical instabilities of the platform itself. Similar calibration-related overestimation has been reported by Schomburg et al. [17], who observed occasional overestimation of load-sensor based weights in a load-cell-based poultry weighing system. Additionally, non-uniform load distribution due to bird posture, leg positioning, and the erratic movement of birds may have affected the force transfer from the platform to individual load cells. The mechanical stability of the platform may have further affected the measurement accuracy, as sensor noises arising from such sources may further introduce systematic error as observed in our system. However, among these factors, calibration is considered the most likely factor contributing to the observed overestimation, since the bias was consistent across birds rather than occurring randomly. Finally, data filtering choices including the rolling-median filter and session-detection algorithm were used by the system to reduce the sensitivity to signal noises; they may also introduce additional bias during short visits where noise may dominate the actual measurement. Future work involving controlled bench testing, multi-point calibration, and isolation of these contributors are required to quantify their contribution and improve systematic accuracy. There is also a potential issue of birds using platform as a resting place, resulting in continuous weight readings from a single bird. However, we used a cut-off threshold of 10 readings per session to avoid such issue.

Another major limitation of our study was that we did not develop a system to identify the number of chickens stepping on the platform simultaneously, which might lead to an overestimation of the weights when multiple birds are stepping on the platform. Previous automated systems have used radio-frequency identification (RFID) and Bluetooth low energy (BLE) tags [17,18,19], which can identify instances of multiple birds and infer accurate weights. Additionally, computer vision-based systems have been developed that work in conjunction with physical weighing systems to identify the number of birds the platform is weighing [20].

In addition to physical sensor-based setups, computer vision techniques alone have also been used to estimate the weight of chickens. For example, Qiao et al. [21] used RGB images captured from a tri-directional camera setup combined with deep learning models to estimate body weight of Pekin ducks, achieving an accuracy of 98.135%. While vision-based methods offer the advantage of contactless measurement and reduced hardware complexity, their accuracy can be influenced by lighting conditions, bird posture, occlusion, and environmental complexities present in commercial housing conditions. In comparison, our system demonstrated higher precision than several reported vision-only approaches. For example, a 2D video analysis system proposed by Campbell et al. [22] yielded an MAPE of 7%, which is notably higher than the error observed in the current sensor-based platform. This highlights the advantage of direct load-cell measurement over some image-derived estimations, while also suggesting that integration of vision-based approaches with physical weighing platforms may offer higher accuracies and a direction for future improvements.

Additionally, our system also offers advantages in terms of cost and scalability, especially in case of large commercial operations. In addition to algorithmic measures, the placement of the weighing platform is also necessary to avoid inaccurate flock weight estimations. Pasian et al. [7] suggested that, to obtain a representative sample of the flock, one scale must be placed in each quadrant of the poultry house. Our system is designed as a low-cost solution in which a single Rpi can wirelessly collect and process data from multiple weighing units. Once the core system is established, additional weighing platforms can be added at low costs, making the proposed approach suitable for large-scale implementation in commercial poultry facilities. Since communication between the platform employing MCU and Rpi relies on wireless transmission, the system may experience occasional data losses due to unstable network conditions, as evident in wireless sensor networks used in precision agriculture scenarios [23]. However, this system also uses a micro-SD card for local data storage, which helps mitigate the impacts of temporary communication and data losses.

Hence, building on these findings and limitations, future work will focus on the proper calibration of the load cells and applying more robust data filtering techniques for accurate weight monitoring in dynamic cage-free environment. Furthermore, the proposed system will be extended to bird-level identification using low-cost wearables and computer vision techniques to count the number of birds on the scale. Incorporating visual information would enable automatic recognition of individual birds or confirmation of full contact with the weighing platform, thereby reducing errors caused by partial loading or simultaneous platform use by multiple birds. Such integration would also allow weight measurements to be linked to individual birds over time, supporting accurate monitoring of growth patterns and health status.

## 4. Conclusions

This study developed and tested an automated weighing platform for monitoring bird weights in cage-free poultry houses. The system combines load-cell sensing, an ESP32 microcontroller for data collection and wireless transmission, and a Raspberry Pi for processing, filtering, and displaying the measurements. The platform successfully detected weight differences between birds and demonstrated improved performance when installed on a stable surface. However, the system had a consistent but small overestimation, likely due to calibration issues and mechanical instability of the platform itself. Despite these limitations, the prototype showed strong potential for stress-free real-time weight monitoring in commercial poultry environments. Future work will focus on improving the platform’s mechanical stability, refining the calibration, strengthening the filtering algorithms, and addressing multi-bird events to improve system performance. Additional studies will examine how birds interact with the platform in commercial conditions, explore bird-level identification strategies, and incorporate edge-based analytics to support flock management and welfare monitoring.

## Figures and Tables

**Table 1:** Estimated cost breakdown of the automated weighing system.

Component	Description	Quantity	Estimated Cost (USD)
Raspberry Pi 5	Single board computer	1	~86
LCD screen	Display unit for displaying system status and live weights	1	~4
ESP32-S3	Microcontroller unit	1	~6
SD card module + SD card	Data storage module	1 each	~6
HX711 amplifier + 4 pcs load cell sensors	Weight registration	1 set	~5
Real time clock module	Module for accurate timestamp	1	3
Square PVC panel	Platform for weighing system	1	~8
TOTAL			~118

## Data Availability

The data presented in this study are available upon reasonable request to the corresponding author.

## Abbreviations

The following abbreviations are used in this manuscript:

ADC	Analog-to-digital converter
BLE	Bluetooth low energy
CSV	Comma-separated values
I2C	Inter-integrated circuit
IACUC	Institutional Animal Care and Use Committee
IoT	Internet of Things
LCD	Liquid crystal display
LOA	Limits of agreement
MAPE	Mean absolute percentage error
MCU	Microcontroller unit
MQTT	Message queuing telemetry transport
PVC	Polyvinyl chloride
RFID	Radio-frequency identification
RGB	Red–green–blue
RTC	Real time clock
SD	Secure digital
UNIX	UNiplexed Information and Computing System
